# Diverticular Stricture and Hepatic Abscess Formation During Tirzepatide Therapy for Obesity

**DOI:** 10.7759/cureus.96564

**Published:** 2025-11-11

**Authors:** Bethany Taylor, Edward Farley-Hills, Wah Yang, Sjaak Pouwels, Graham Whiteley, Danielle Wilkinson, Anurag Agarwal, Mahmoud Al-Ardah, Andrew Beamish, Mohammed Hammoda, Rebecca V Hoffmann, Ahmed Ahmed, Suhaib Ahmad

**Affiliations:** 1 Department of Anaesthesia and Intensive Care, Betsi Cadwaladr University Health Board, Bangor, GBR; 2 Department of Surgery, The First Affiliated Hospital of Jinan University, Guangzhou, CHN; 3 Department of Intensive Care Medicine, Elisabeth-Tweesteden Hospital, Tilburg, NLD; 4 Department of General Surgery, Betsi Cadwaladr University Health Board, Bangor, GBR; 5 Department of General Surgery, Health Education and Improvement Wales, Swansea, GBR; 6 Department of Pharmacology, Coventry University, Coventry, GBR; 7 Department of General Surgery, Imperial College London, London, GBR

**Keywords:** bacterial translocation, diverticular disease, gastrointestinal complications, glp-1 receptor agonist, hepatic abscess, obesity pharmacotherapy, rapid weight loss, tirzepatide therapy, type 2 diabetes, weight management

## Abstract

Tirzepatide, a dual glucose-dependent insulinotropic polypeptide (GIP)/glucagon-like peptide-1 (GLP-1) receptor agonist, is increasingly used worldwide for weight loss and glycaemic control but is associated with gastrointestinal side effects. We hereby present a case of a complicated diverticulitis with hepatic abscesses in a patient with pre-existing asymptomatic diverticular disease on tirzepatide. The patient, a 53-year-old man, developed severe sepsis and multi-organ dysfunction following rapid 25 kg weight loss. Imaging revealed sigmoid stricture, obstructive diverticulitis, and liver abscesses. Proposed mechanisms include reduced colonic motility, altered bile flow, and microbiota changes induced by tirzepatide, exacerbating diverticular disease and promoting bacterial translocation. This case highlights the need for clinical vigilance in patients with known or incidental diverticular disease receiving GLP-1 based therapies. Further research is warranted to assess whether routine monitoring should be implemented in this potentially high-risk subgroup.

## Introduction

Obesity is a complex, multifactorial disease influenced by genetic, behavioural, and environmental factors. It results from an imbalance between energy intake and expenditure, leading to excessive fat accumulation that impairs health. The condition is classified based on body mass index (BMI), with a BMI ≥30 kg/m² defining obesity. Beyond its metabolic and cardiovascular consequences, obesity contributes to chronic inflammation and organ dysfunction, underscoring its recognition as a global epidemic. Advances in understanding the hormonal regulation of appetite and glucose metabolism have driven the development of incretin-based therapies, which now play a central role in both diabetes and obesity management. Obesity remains one of the most common long-term health conditions, currently affecting over 650 million adults worldwide. It is linked with serious complications such as type 2 diabetes, cardiovascular disease, and premature mortality, while also creating a major economic strain on global healthcare systems [1-3].

Although lifestyle modification is the first step in managing obesity, lasting weight reduction is often difficult to achieve because of strong physiological mechanisms that resist weight loss [4]. In recent years, pharmacological therapy has become an increasingly important addition to lifestyle measures for patients with obesity and related comorbidities [5].

Tirzepatide is a new dual incretin receptor agonist that targets both the glucose-dependent insulinotropic polypeptide (GIP) and glucagon-like peptide-1 (GLP-1) pathways. Initially approved for the treatment of type 2 diabetes, it has also shown remarkable effects on weight reduction in clinical trials [6,7]. By stimulating both incretin receptors, it produces complementary improvements in glycaemic control and body-weight regulation.

While the metabolic benefits of tirzepatide are well recognised, gastrointestinal adverse events, including nausea, vomiting, constipation, and biliary disorders, have been frequently observed. There are also isolated reports of pancreatitis, hepatobiliary dysfunction, and intestinal obstruction. However, there is limited evidence on the potential risks in patients with pre-existing gastrointestinal disease such as diverticulosis or on rare complications like hepatic abscesses [8-10].

We report a case of severe sepsis with complicated diverticulitis and hepatic abscesses in a patient receiving tirzepatide for weight reduction. This case raises awareness of possible gastrointestinal and hepatobiliary complications related to rapid weight loss and provides insight into potential mechanisms for clinicians to consider.

## Case presentation

A 53-year-old man presented to the emergency department with a three-day history of malaise, jaundice, and fever. His medical history reported type 2 diabetes mellitus and hypertension. Over the preceding five months, he took tirzepatide for weight loss and successfully lost 25 kg. He reported no recent travel, no family or personal history of liver or biliary disease, and no alcohol or recreational drug use.

During the first three months of therapy, the dose was increased from 2.5 mg to 10 mg weekly and then reduced to 2.5 mg over the following two months.

On arrival, he was hypotensive, tachycardic, pyrexial, and visibly jaundiced. Clinical examination revealed a soft, tender epigastrium. Initial arterial blood gas analysis demonstrated compensated metabolic acidosis with hyperlactataemia. Laboratory investigations are summarised in Table 1, showing markedly raised inflammatory markers, renal and hepatic dysfunction, thrombocytopenia, and elevated serum amylase.

**Table 1:** Laboratory results on admission.

Parameter	Result	Reference Range	Unit
White cell count (WCC)	57.9	4.0 – 11.0	× 10⁹/L
C-reactive protein (CRP)	132	< 5	mg/L
Amylase	2208	30 – 110	U/L
Bilirubin (peak)	207	< 21	µmol/L
Alanine aminotransferase (ALT, peak)	1661	< 45	U/L
CA 19-9	6606	< 37	kU/L

Computed tomography (CT) abdomen and pelvis revealed moderate intrahepatic biliary dilatation and suspected cholangitis, multifocal hepatic hypodensities, an indeterminate lesion in the left kidney, and severe sigmoid diverticulosis with incomplete large bowel obstruction. 

In view of severe sepsis and multi-organ dysfunction, the patient was transferred to the intensive care unit (ICU), where continuous venovenous hemodiafiltration (CVVHDF) was initiated for anuria and worsening acidosis. Liver function tests deteriorated further, with a peak bilirubin of 207 µmol/L and alanine aminotransferase (ALT) rising to 1661 U/L. Tumour markers were elevated, with carbohydrate antigen 19-9 (CA 19-9) measuring 6606 kU/L.

Bedside ultrasound demonstrated a heterogeneous liver with an oedematous gallbladder and mildly dilated common bile duct, without clear intraluminal obstruction. An initial upper GI multidisciplinary team (MDT) discussion (20/02/2025) recommended percutaneous transhepatic cholangiography and biliary drainage (booked for 21/02/2025), with consideration of subsequent endoscopic retrograde cholangiopancreatography; however, this plan was delayed due to worsening coagulopathy requiring multiple blood product transfusions. Despite this, the patient maintained his own airway with high-flow oxygen and required only minimal vasopressor support, which was weaned by the end of the first ICU week.

**Figure 1:**
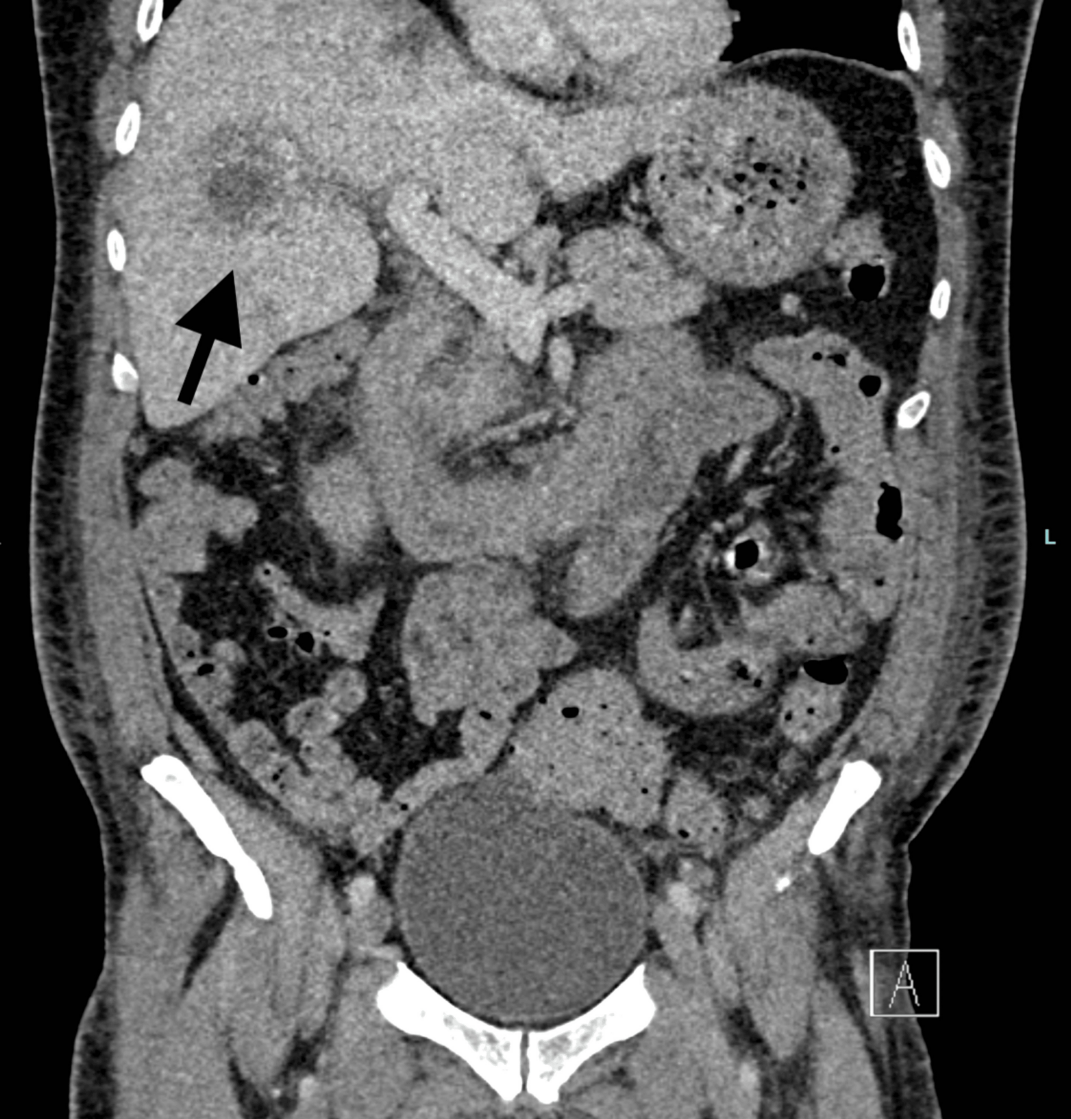
Contrast-enhanced CT abdomen demonstrating multiple low-attenuation lesions within the right hepatic lobe, consistent with evolving hepatic abscesses. The distribution and appearance support portal venous spread from an intra-abdominal source, in keeping with polymicrobial sepsis.

Blood cultures yielded Klebsiella, Clostridium perfringens, and Escherichia coli, with a procalcitonin level >100 confirming severe bacterial sepsis. A repeat CT scan on 7/03/2025 revealed multiple hepatic low-density lesions consistent with evolving liver abscesses (Figure 1).

As his condition gradually stabilised, CVVHDF was discontinued, intermittent haemodialysis commenced, and liver function slowly improved. Magnetic resonance cholangiopancreatography was requested to evaluate the biliary tree further, but it had to be cancelled due to the presence of a previous metallic foreign body, contraindicating magnetic resonance imaging (MRI).

By the third week of ICU admission, with improvement in coagulopathy and infection markers, and with renal function no longer dialysis-dependent, the patient was discharged to the ward. A repeat CT demonstrated heterogeneous liver texture with two residual fluid collections (36 mm and 30 mm), likely representing maturing hepatic abscesses. The gallbladder remained oedematous, but there was no biliary obstruction.

**Figure 2:**
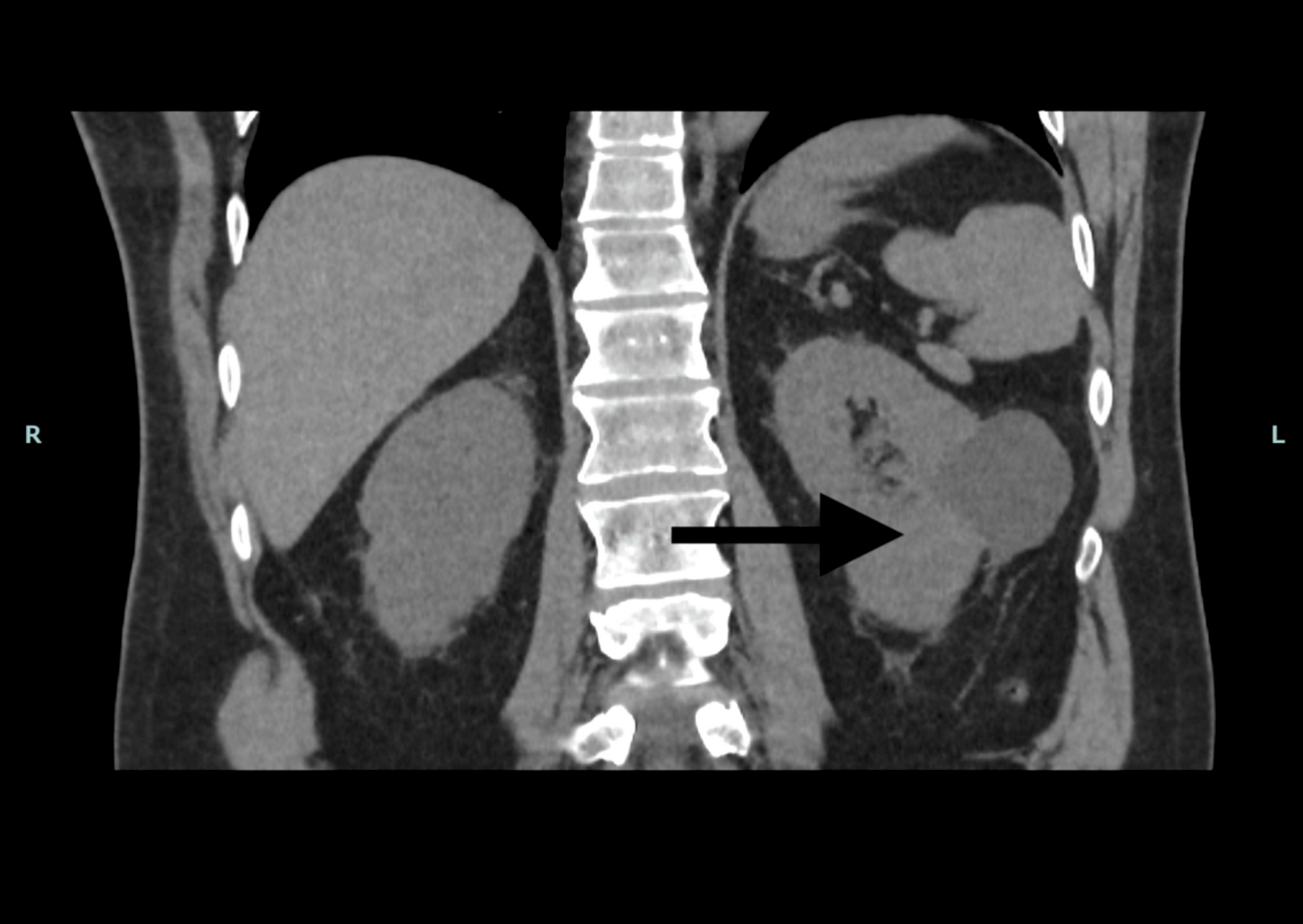
CT kidney showing an indeterminate left renal lesion with features suggestive of a haemorrhagic cyst. No enhancing or infiltrative characteristics were observed, supporting a benign process.

During this period, the patient was also referred to the Urology MDT (19/03/2025) due to the indeterminate left renal lesion noted on earlier CT. The lesion remained unchanged, favouring a benign process such as a haemorrhagic cyst, but a three-phase CT kidney scan with contrast was recommended, with follow-up in three months. This plan was discussed again at the urology clinic (04/04/2025), where the patient remained asymptomatic (Figure 2).

**Figure 3:**
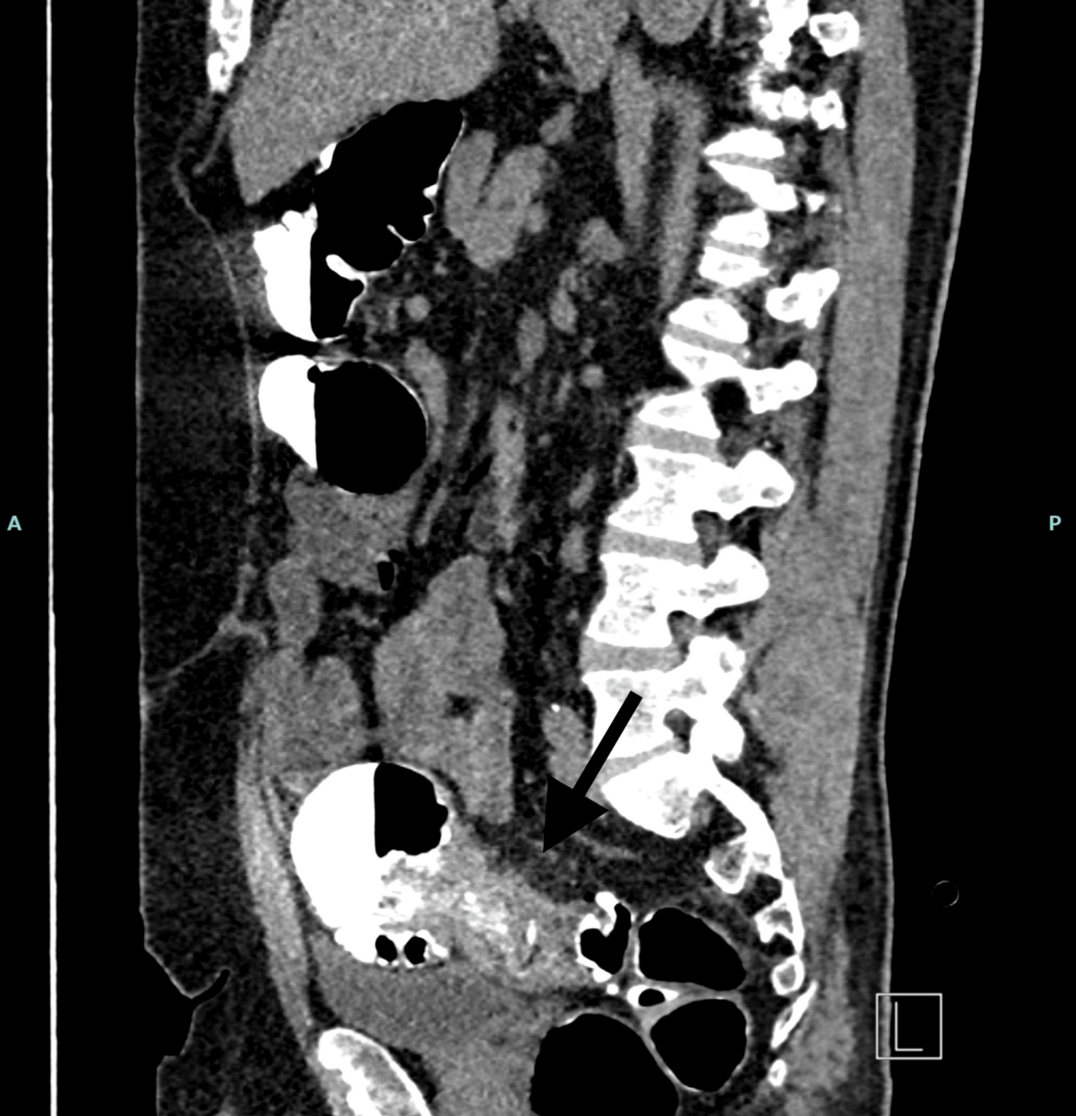
CT colonography revealing a circumferential shouldered lesion within the sigmoid colon, proximal to a segment of severe diverticular disease. The narrowing and mural thickening correspond to the fibrotic stricture identified intraoperatively, consistent with chronic diverticulitis rather than malignancy.

A flexible sigmoidoscopy was attempted prior to discharge but could not be completed due to a circumferential stricture at 30 cm, raising concerns about an underlying malignancy. CT colonography performed on 30/04/2025 showed apparent resolution of the liver abscesses but identified a suspicious shouldered lesion in the sigmoid colon proximal to a segment of severe diverticular disease. This case was subsequently reviewed at the lower GI MDT (09/05/2025). The MDT recommended elective sigmoid resection, given the suspicious nature of the lesion and the patient’s prior septic presentation (Figure 3).

Following a four-week total inpatient stay, the patient was discharged home with outpatient follow-up under general surgery and urology.

Subsequently, the patient underwent elective open sigmoid resection. Histological analysis of the resected specimen demonstrated complex diverticular disease with muscularis propria hypertrophy and fibrosis of the submucosa. No evidence of dysplasia, malignancy, or acute abscess formation was seen. There was some acute serositis of uncertain significance. Nine lymph nodes were sampled, all negative for malignancy. No colonic mass was identified, and the luminal narrowing was attributed to fibrosis and chronic inflammation secondary to diverticular disease.

At the final lower GI MDT review on 6 June 2025, there was no evidence of malignancy, and the patient was discharged from further multidisciplinary follow-up.

Outcome and follow-up

Histological analysis of the resected specimen confirmed complex diverticular disease with muscular hypertrophy and fibrosis. There was no evidence of dysplasia or malignancy. Nine lymph nodes were examined, all negative for malignancy.

At the follow-up, the patient’s renal function had recovered sufficiently to be independent of dialysis, and liver function tests showed continued improvement. Imaging confirmed resolution of hepatic abscesses. The renal lesion remained unchanged and was considered benign, with surveillance imaging planned at three months.

At the multidisciplinary review, the patient was discharged from oncology and surgical cancer surveillance. He has since returned home and is gradually resuming daily activities.

The key diagnostic clues in this case included rapid, medication-associated weight loss, marked sepsis with polymicrobial bacteremia, and imaging findings of hepatic abscesses in the context of severe diverticular disease. The absence of biliary obstruction despite cholestatic liver dysfunction, along with the temporal association with tirzepatide use, helped distinguish this presentation from primary biliary or malignant processes. 

## Discussion

This case describes a rare combination of complicated diverticulitis, hepatic abscesses, and biliary dysfunction in a patient treated with tirzepatide for obesity. Although a direct causal relationship cannot be proven, the timing of events suggests that tirzepatide and the rapid weight loss it induced may have played a contributory role.

Diverticulitis develops when structural changes in the colonic wall, such as fibrosis and mucosal weakness, allow local inflammation and bacterial overgrowth. Increased intraluminal pressure and reduced motility are key factors in this process [11]. GLP-1 receptor agonists are known to slow gastric and intestinal transit, which can further raise colonic pressure and potentially trigger inflammation in susceptible individuals [12].

Rapid weight reduction itself can modify bile composition and gut microbiota. These changes may impair mucosal integrity, promoting bacterial translocation and, in severe cases, systemic infection [13,14]. In our patient, these mechanisms could explain the progression from diverticulitis to hepatic abscess formation. The organisms isolated, Klebsiella, E. coli, and Clostridium perfringens, are consistent with portal venous spread from an intra-abdominal source [15].

The markedly elevated CA 19-9 was interpreted as a non-specific inflammatory response rather than a tumour-driven rise, as there was no radiological or histological evidence of malignancy. Similarly, the elevated amylase level most likely reflected systemic inflammation rather than acute pancreatitis, since no pancreatic abnormalities were identified on imaging.

It is also worth noting that tirzepatide is often titrated more aggressively in weight management than in diabetes care, which might amplify gastrointestinal side effects. This may partly explain the severity of complications in this patient.

Clinicians should be aware that patients with known or incidental diverticular disease may represent a higher-risk group when starting GLP-1-based therapy. Although current guidelines do not recommend screening for diverticular disease before initiation, a more cautious approach and closer monitoring may be appropriate when rapid weight loss is anticipated.

Although an association existed between tirzepatide use and this patient’s presentation, causation cannot be definitively established. The patient had not recently initiated tirzepatide and was on a reduced maintenance dose at the time of admission, which weakens a direct temporal link. Other contributing factors, such as pre-existing diverticular disease, diabetes, rapid weight loss, and possible alterations in gut motility or microbiota, may have collectively predisposed to infection and inflammation.

Learning points

Tirzepatide, a dual GLP-1 and GIP receptor agonist, may precipitate serious gastrointestinal complications in patients with pre-existing diverticular disease. Rapid weight loss and altered gut motility associated with its use can promote bacterial translocation and infection, occasionally leading to severe sequelae such as hepatic abscess formation. Clinicians should therefore maintain a high index of suspicion and consider proactive risk assessment and monitoring when initiating GLP-1-based therapies in individuals with known or incidental diverticular disease.

## Conclusions

This case demonstrates a rare but clinically important sequence of complications, hepatic abscesses, biliary dysfunction, and complicated diverticulitis, occurring after tirzepatide-associated rapid weight loss. Although causation cannot be confirmed, the temporal relationship suggests that tirzepatide may contribute to gastrointestinal and hepatobiliary events in predisposed patients.

Careful patient selection and monitoring are essential, particularly for those with known or incidental diverticular disease. Further research is required to clarify underlying mechanisms and to establish evidence-based guidance for managing patients who develop gastrointestinal complications during incretin-based therapy.
